# Editorial: Strength training and performance optimization: the triad of physical, psychological, and physiological excellence

**DOI:** 10.3389/fpsyg.2026.1791628

**Published:** 2026-02-24

**Authors:** Henrique P. Neiva, David Rodríguez-Rosell, Ana Pereira

**Affiliations:** 1 Department of Sport Sciences, University of Beira Interior, Covilhã, Portugal; 2 Research Center in Sports Sciences, Health Sciences and Human Development, CIDESD, Covilhã, Portugal; 3 Department of Sport and Computer Science, Universidad Pablo de Olavide, Sevilla, Spain; 4 Physical Performance & Sports Research Center, Universidad Pablo de Olavide, Sevilla, Spain; 5 Research, Development and Innovation (R&D+i) Area, Investigation in Medicine and Sport Department, Sevilla Football Club, Seville, Spain; 6 Instituto Politécnico de Setúbal, Escola Superior de Educação, Setúbal, Portugal; 7 Sport Physical Activity and Health Research and Innovation Center, Rio Maior, Portugal

**Keywords:** acute effects, chronic effects, muscle strength, physical exertion, resistance training

Strength training is essential to modern methods for enhancing performance, but several scientific and practical issues remain unclear. Progress in this area requires a better understanding of how strength and power adaptations affect the physiological, biomechanical, and psychological factors that support athletic performance. Improvements in force output, movement speed, and rate of force development are crucial for winning, yet the mechanisms behind these changes and how they interact with training variables need more research. This Research Topic brings together contributions that address these gaps by examining acute and chronic effects of strength and resistance training, analyzing technical and neuromuscular determinants of performance, and highlighting the role of psychological and perceptual factors.

A first group of studies in this Research Topic highlights the significance of psychological and perceptual factors in explaining performance outcomes. Herzberg et al. examined how manipulated task duration affects perceived exertion and fatigue during an isometric endurance task in adults aged 25–50. Participants who experienced an unexpected prolongation of the task reported increased perceived exertion without corresponding rises in muscular fatigue. Interestingly, those with lower fatigue levels were more vulnerable to this manipulation. The authors proposed that ratings of perceived exertion during endurance tasks reflect the level of physiological fatigue-related parameters but do not predict task failure, emphasizing that psychological regulation plays a key role in exercise tolerance. Discussing these perceptual dimensions, Torres et al. synthesized research on perceived exertion across continuous sports, identifying variability in methods and underrepresentation of female athletes. Their results showed that the menstrual cycle phase influences perceptual load and physiological responses, raising important considerations for sex-specific monitoring and training. This review also highlights the lack of studies on older athletes, despite their growing participation. Both articles demonstrate the need to standardize perceived exertion guidelines and adopt more inclusive research designs.

Research on performance optimization emphasizes that physical training programs should not only reduce injury and disease risk but also target modifiable factors. Li et al. provide deeper insight into individual risk factors among individuals who completed physical detoxification in China's mandatory isolation drug rehab centers. Although this setting differs from traditional sports environments, the study reveals how drug type, sex differences, and cognitive factors influence physical activity, fitness levels, and psychological craving. Their findings have broader implications for exercise prescription in vulnerable groups: low-intensity aerobic training with progressive resistance may reduce physiological dependence, while high-intensity interval training combined with cognitive tasks can enhance cognitive regulation. This work highlights the interplay of exercise, performance, physiological, and psychosocial factors.

Providing deeper insight into how individuals regulate intensity during exercise training, Almeida et al. highlighted the manipulation of volume and intensity to induce different stimuli. They compared the effects of Rest-Pause Training (sets performed with small intra-set pauses of 20 s after concentric failure), Sarcoplasmic Stimulating Training (sets performed with intra-set pauses of 20 s after concentric failure and then a final set to concentric failure with a 20% intensity reduction), and a traditional session (sets performed until concentric failure with 1 min rest between) in metabolic and psycho-affective responses in adult men (*n* = 15). Their findings suggest that individuals with higher training levels may benefit from increased repetition volumes to maximize adaptation, while coaches should consider psycho-affective responses when prescribing workloads. Complementing this perspective, Páez-Maldonado et al. examined mechanical performance and neuromuscular fatigue across various set configurations in the full squat. It is suggested that cluster sets improve mechanical performance and show reduced neuromuscular alterations throughout repetitions compared to traditional sets. Therefore, the authors recommend that strength and conditioning coaches consider integrating short intra-set rest periods, such as 30 s, into training sessions to maintain velocity and reduce fatigue buildup.

Addressing safety in resistance training, Rojas-Jaramillo et al. conducted a scoping review on the impact of deep squats on knee osteoarticular health. Their synthesis indicated that the deep squat is safe for knee joint health and can be included in resistance programs for individuals without prior pathologies, provided proper technique is used. They note that evidence remains limited and call for long-term studies to clarify this Research Topic and dispel the belief that greater knee flexion increases osteoarticular risk. Additionally, anatomical characteristics, such as joint properties and range of motion, influence performance. Larsen et al. examined the effect of range of motion on gastrocnemius hypertrophy in untrained men, reporting that peak dorsiflexion with partial repetitions beyond failure may enhance hypertrophic adaptation, offering insights for programming strategies extending time under tension. Together, these studies highlight how exercise technique and repetition structure modulate neuromuscular outcomes and long-term adaptations.

Another thematic axis of this Research Topic relates to the biomechanical determinants of explosive performance. Yoshida et al. compared the kinetic variables of the three lower limb joints during take off that affect the drop height and the jump index of a drop jump from different drop heights (0.3, 0.6, and 0.9 m) in male athletes. They found that the drop height in the jump index and the rebound jump index are influenced by the torque, power, and work, and vary with the drop height. This illustrates the importance of mechanical profile and reinforces that plyometric training must account for the kinetic demands of explosive movements.

Finally, several contributions in this Research Topic address the specificity of training and performance optimization in particular sports. The impact of the sport, different training methods, and evaluation tools have been explored in this Research Topic. For example, Zhu and Wang examined the overall performance of top male and female athletes in the 2020 Tokyo Olympics in trampoline gymnastics, following the implementation of the “Horizontal New Rule.” With the implementation of this rule, the authors observed that the timing of flight had the greatest influence on total score for men, followed by difficulty and execution. On the other hand, in females, difficulty dominated, followed by timing and horizontal, with execution least impactful. The authors noted that it is essential to increase strength and explosiveness, gradually raise movement difficulty and jump height, and advance technology to develop a more solid, evidence-based theoretical framework for training design.

In aquatic sports, Yang et al. studied the effects of anodal transcranial direct current stimulation (a-tDCS) on 24 top-level freestyle swimmers from China. The athletes who received a-tDCS combined with physical training showed improvements in basic strength, explosiveness, and anaerobic endurance. Surprisingly, the a-tDCS technique did not enhance or relate to aerobic endurance. This highlights that much remains to be understood about the anatomical and physiological adaptations to training and their connection to performance. Supporting this, Raineteau et al. examined the training and testing practices of Strength and Conditioning coaches in the French Swimming Federation and found significant gaps in the scientific understanding of testing and monitoring various parameters in dry-land and swimming contexts. The authors encourage coaches to deepen their understanding, utilize user-friendly and affordable evaluation technologies, and adopt spatio-temporal analysis in swimming.

Taken together, this Research Topic provides a multifaceted perspective on strength training and performance optimization. Performance emerges from the interplay between physical, physiological, and psychological dimensions, and effective training prescription requires integrating perceptual regulation, neuromuscular responses, biomechanical determinants, and sport-specific adaptations.

